# Contest models highlight inherent inefficiencies of scientific funding competitions

**DOI:** 10.1371/journal.pbio.3000065

**Published:** 2019-01-02

**Authors:** Kevin Gross, Carl T. Bergstrom

**Affiliations:** 1 Department of Statistics, North Carolina State University, Raleigh, North Carolina, United States of America; 2 Department of Biology, University of Washington, Seattle, Washington, United States of America; Stanford University School of Medicine, UNITED STATES

## Abstract

Scientific research funding is allocated largely through a system of soliciting and ranking competitive grant proposals. In these competitions, the proposals themselves are not the deliverables that the funder seeks, but instead are used by the funder to screen for the most promising research ideas. Consequently, some of the funding program's impact on science is squandered because applying researchers must spend time writing proposals instead of doing science. To what extent does the community's aggregate investment in proposal preparation negate the scientific impact of the funding program? Are there alternative mechanisms for awarding funds that advance science more efficiently? We use the economic theory of contests to analyze how efficiently grant proposal competitions advance science, and compare them with recently proposed, partially randomized alternatives such as lotteries. We find that the effort researchers waste in writing proposals may be comparable to the total scientific value of the research that the funding supports, especially when only a few proposals can be funded. Moreover, when professional pressures motivate investigators to seek funding for reasons that extend beyond the value of the proposed science (e.g., promotion, prestige), the entire program can actually hamper scientific progress when the number of awards is small. We suggest that lost efficiency may be restored either by partial lotteries for funding or by funding researchers based on past scientific success instead of proposals for future work.

## Abstract 2

Scientists waste substantial time writing grant proposals, potentially squandering much of the scientific value of funding programs. This Meta-Research Article shows that, unfortunately, grant-proposal competitions are inevitably inefficient when the number of awards is small, but efficiency can be restored by awarding funds through a modified lottery, or by weighting past research success more heavily in funding decisions.

## Author summary

The grant proposal system compels researchers to devote substantial time to writing proposals that could have instead been used to do science. Here, we use the economic theory of contests to show that as fewer grants are funded, the value of the science that researchers forgo while preparing proposals can approach or exceed the value of the science that the funding program supports. As a result, much of the scientific impact of the funding program is squandered. Unfortunately, increased waste and reduced efficiency is inevitable in a grant proposal competition when the number of awards is small. How can scarce funds be allocated efficiently, then? As one alternative, we show that a partial lottery that selects proposals for funding randomly from among those that pass a qualifying standard can restore lost efficiency by reducing investigators' incentives to invest heavily in preparing proposals. Lotteries could also improve efficiency by compelling administrators to de-emphasize grant success as a primary measure of professional achievement. If lotteries are politically untenable, another remedy would be to fund researchers based on their previous research successes, although in such a way that avoids establishing barriers to entry for junior scientists or scientists from historically underrepresented demographic groups.

## Introduction

Over the past 50 years, research funding in the United States has failed to keep pace with growth in scientific activity. Funding rates in grant competitions have plummeted (S1 Fig, [1–4]) and researchers spend far more time writing grant proposals than they did in the past [5]. A large survey of top US universities found that, on average, faculty devote 8% of their total time—and 19% of their time available for research activities—towards preparing grant proposals [6]. Anecdotally, medical school faculty may spend fully half their time or more seeking grant funding [5, 7]. While the act of writing a proposal may have some intrinsic scientific value [8]—perhaps by helping an investigator sharpen ideas—much of the effort given to writing proposals is effort taken away from doing science [9]. With respect to scientific progress, this time is wasted [10].

Frustrated with the inefficiencies of the current funding system, some researchers have called for an overhaul of the prevailing funding model [9, 11–18]. In particular, Fang and Casadevall [16] recently suggested a partial lottery, in which proposals are rated as worthy of funding or not, and then a subset of the worthy proposals are randomly selected to receive funds. Arguments in favor of a partial lottery include reduced demographic and systemic bias, increased transparency, and a hedge against the impossibility of forecasting how scientific projects will unfold [16]. Indeed, at least three funding organizations—New Zealand's Health Research Council and their Science for Technological Innovation program, as well as the Volkswagen Foundation [19]—have recently begun using partial lotteries to fund riskier, more exploratory science.

Compared with a proposal competition, a lottery permits more proposals to qualify for funding and thus lowers the bar that applicants must clear. A lottery also offers a lower reward for success, as a successful proposal receives a chance at funding, not a guarantee of funding. Thus, we expect that investigators applying to a partial lottery will invest less time and fewer resources in writing a proposal. To a first approximation, then, a proposal competition funds high-value projects while wasting substantial researcher time on proposal preparation, whereas a partial lottery would fund lower-value projects on average but would reduce the time wasted writing proposals. It is not obvious which system will have the greater net benefit for scientific progress.

In this article, we study the merits and costs of traditional proposal competitions versus partial lotteries by situating both within the rich economic theory of contests. In this theory, competing participants make costly investments ("bids") in order to win one or more prizes [20, 21]. Participants differ in key attributes, such as ability and opportunity cost, that determine their optimal strategies. In an economics context, contests are often used by the organizer as a mechanism to elicit effort from the participants. For example, TopCoder and Kaggle are popular contest platforms for tech firms (the organizers) to solicit programming or data-analysis effort from freelance workers (the participants). However, because the participants' attributes influence their optimal strategies, the bids that participants submit reveal those attributes. Thus, screening of participants often arises as a side effect.

In funding competitions, the organizer is a funding body and the participants are competing investigators. Investigators pitch project ideas of varying scientific value by preparing costly proposals. However, unlike a traditional economic contest, the funding body's primary objective is to identify the most promising science, using proposals to screen for high-value ideas. The funding body has little interest in eliciting work during the competition itself, as the proposals are not the deliverables the funder seeks. All else equal, the funding agency would prefer to minimize the work that goes into preparing proposals, to leave as much time as possible for investigators to do science. In this case, how should the funder organize the contest to support promising science without squandering much of the program's benefit on time wasted writing proposals?

Below, we pursue this question by presenting and analyzing a contest model for scientific funding competitions. We first use the model to assess the efficiency of proposal competitions for promoting scientific progress and ask how that efficiency depends on how many proposals are funded. We next explore how efficiency is impacted when extrascientific incentives such as professional advancement motivate scientists to pursue funding, and compare the efficiency of proposal competitions with that of partial lotteries. Finally, we reflect on alternative ways to improve the efficiency of funding competitions without adding intentional randomness to the award process. All of our analyses focus on equilibrium behavior and thus pertain most directly to long-standing funding competitions, for which researchers can acquire experience that informs their future actions.

## A contest model for scientific funding competitions

Our model draws upon a framework for contests developed by Moldovanu and Sela [20]. In our application, a large number of scientists (or research teams) compete for grants to be awarded by a funding body. The funder can fund a proportion *p* of the competing investigators. We call *p* the payline, although *p* could be smaller than the proportion of investigators who are funded if some investigators do not enter the competition.

Project ideas vary in their scientific value, which we write as *v*, where *v*≥0. In this case, scientific value combines the abilities of the investigator and the promise of the idea itself. Although we do not assign specific units to *v*, scientific value can be thought of as some measure of scientific progress, such as the expected number of publications or discoveries. We assume that the funder seeks to advance science by maximizing the scientific value of the projects that it funds, minus the value of the science that investigators forgo while writing proposals. However, the funder cannot observe the value of a project idea directly. Instead, the funder evaluates proposals for research projects, and awards grants to the top-ranked proposals. Assume that proposals can be prepared to different strengths, denoted *x*≥0, with a larger value of *x* corresponding to a stronger proposal. A scientist with a project idea of value *v* must decide how much effort to invest in writing a proposal, that is, to what strength *x* her proposal should be prepared. In our model, this decision is made by a cost–benefit optimization.

On the benefit side, if a proposal is funded, the investigator receives a reward equal to the scientific value of the project, or *v*. This reward is public, in the sense that it benefits both the investigator and the funder. Receiving a grant may also bestow an extrascientific reward on the recipient, such as prestige, promotion, or professional acclaim. Write this extrascientific reward as *v*_0_≥0. This extrascientific reward is private, as it benefits only the grant recipient and not the funder. Let *η*(*x*) be the equilibrium probability that a proposal of strength *x* is funded; *η*(*x*) will be a nondecreasing function of *x*. Thus, in expectation, an investigator with a project of value *v* who prepares a proposal of strength *x* receives a benefit of (*v*_0_+*v*)*η*(*x*).

Preparing a grant proposal also entails a disutility cost equal to the value of the science that the investigator could have produced with the time and resources invested in writing. Let *c*(*v*,*x*) give the disutility cost of preparing a proposal of strength *x* for a project of value *v*. Here, we study the case where *c*(*v*,*x*) is a separable function of *v* and *x*, so we set *c*(*v*,*x*) = *g*(*v*)*h*(*x*). Proposal competitions are effective screening devices because it is easier to write a strong proposal about a good idea than about a poor one. Therefore, *g*(*v*) is a decreasing function of *v*, i.e., *g*′(*v*)<0. For a given idea, it takes more work to write a stronger proposal, and thus *h*′(*x*)>0. Finally, we assume that preparing a zero-strength proposal is tantamount to opting out of the competition, which can be done at zero cost. Thus, *h*(0) = 0.

Preparing a proposal has some scientific value of its own through the sharpening of ideas that writing a proposal demands [8]. Let *k*∈[0,1) be the proportion of the disutility cost *c*(*v*,*x*) that an investigator recoups by honing her ideas. We call the recouped portion of the disutility cost the intrinsic scientific value of writing a proposal. The portion of the disutility cost that cannot be recouped is scientific waste.

**Fig 1:**
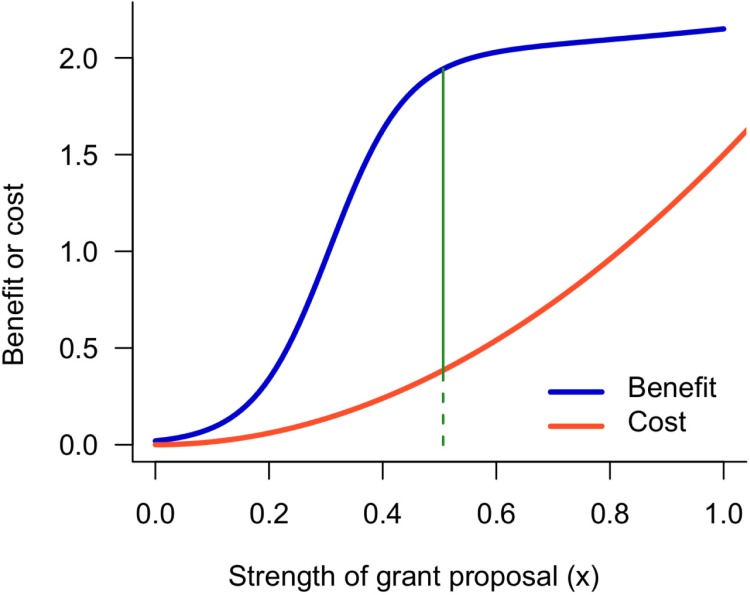
An investigator prepares her grant proposal to the strength that maximizes her payoff. The blue curve shows the expected benefit to the investigator, which is determined by the project's value, any extrascientific reward that the investigator receives from getting the grant, the probability of receiving funding, and the intrinsic value of writing a proposal. The red curve shows the disutility cost of preparing a proposal. The investigator's payoff is the difference between the benefit and the cost. The vertical line shows the bid (Eq 1)—the proposal strength that maximizes the payoff. At the bid, the ratio of the payoff (given by the length of the solid vertical line) to the cost (given by the length of the dashed vertical line) gives the investigator's return on her investment.

All told, the total benefit to the investigator of preparing a proposal to strength *x* is (*v*_0_+*v*)*η*(*x*)+*kc*(*v*,*x*), and the total cost is *c*(*v*,*x*). The difference between the benefit and the cost is the investigator's payoff. The investigator's optimal proposal (or, in economic terms, her "bid") maximizes this payoff (Fig 1):

$$b(v)=\underset{x}{arg\;max}\left\{\left(v_{0}+v)\eta (x)-(1-k)c(v,x\right)\right\}.$$ (1)

For simplicity, we assume that variation among projects is captured entirely in the distribution of *v*, which we write as *F*(*v*). We assume that *v*_0_ and *k* have common values shared by all investigators. In S1 Text we show that our results extend to cases where *v*_0_ or *k* vary among investigators, as long as they are perfectly correlated with *v*.

The challenge in finding the payoff-maximizing bid *b*(*v*) is that the equilibrium probability of funding, *η*(*x*), must be determined endogeneously, in a way that is consistent with both the payline *p* and the distribution of bids that investigators submit. In S1 Text, we follow Hoppe and colleagues [22] to show that, at equilibrium, the bid function is given by

$$b(v)=h^{-1}\left[\frac{1}{1-k}\int_{0}^{v}\frac{v_{0}+t}{g(t)}\xi^{\prime }(t)dt\right].$$ (2)

In Eq 2, *ξ*(*v*) = *η*(*b*(*v*)) is the equilibrium probability that an idea of value *v* is funded. The particular form of *ξ*(*v*) depends on how much randomness is introduced during the review process, which we discuss below.

By comparison, Moldovanu and Sela [20] considered a contest with a small number of competitors, in which the contest's judges observe *x* directly. In their setup, each contestant is uncertain about the strength of her competition (that is, her competitors' types, *v*), but she can be certain that the strongest bid will win the top prize. In our case, we assume that the applicant pool is large enough that the strength of the competition (i.e., the distribution of *v* among the applicants) is predictable. However, the funding agency does not observe *x* directly, but instead convenes a review panel to assess each proposal's strength. Variability among reviewers' opinions then introduces an element of chance into which proposals get funded.

### Scientific efficiency

We use the model to explore how efficiently the grant competition advances science. From the perspective of an individual investigator, the investigator's return on her investment (ROI) is the ratio of her payoff to the cost of her bid:

$$Investigator^{\prime }s\;ROI=\frac{\left(v_{0}+v)\eta (b(v))-(1-k)c(v,b(v)\right)}{c(v,b(v))}.$$ (3)

An investigator will never choose to write a proposal that generates a negative payoff, because she can always obtain a payoff of 0 by opting out. (If the investigator opts out, Eq 3 evaluates to 0/0, in which case we define her ROI to be 0.) Thus, an investigator's equilibrium ROI must be ≥0.

To analyze the funding program's impact on scientific progress as a whole, we compare the total value of the science that the funding program supports with the total value of the science that has been squandered preparing proposals. Of course, both of these quantities will be confounded with the number of grants that are funded, so we standardize to a per-funded-proposal basis. In notation, the average scientific value per funded proposal is

$$\frac{1}{p}\int v\eta (b(v))dF(v),$$ (4)

and the average scientific waste per funded proposal is

$$\frac{1}{p}\int (1-k)c(v,b(v))dF(v).$$ (5)

We will refer to the difference between these two quantities as the scientific gain (or loss, should it be negative) per funded proposal, which is our measure of the funding program's scientific efficiency.

Note that while an investigator will never enter a grant competition against her own self interest, there is no guarantee that the scientific value per funded proposal will exceed the scientific waste. This is because the investigator's payoff includes private, extrascientific rewards obtained by winning a grant (*v*_0_), and (in our accounting, at least) these extrascientific rewards do not benefit the funding agency. If extrascientific motivations for winning grants are large enough, investigators may enter a grant competition even when doing so decreases their scientific productivity. If enough investigators are motivated accordingly, then the scientific progress sacrificed to writing proposals could exceed the scientific value of the funding program. In this case, the grant competition would operate at a loss to science, and the funding agency could do more for science by eschewing the proposal competition and spreading the money evenly among active researchers in the field, or by giving the money to researchers selected entirely at random.

## Analysis and numerical results

We illustrate the model's behavior by choosing a few possible sets of parameter values. Our parameter choices are not directly informed by data. Thus, while the numerical examples illustrate the model's possible behavior, we highlight the results that are guaranteed to hold in general. Throughout, we use the following baseline set of parameters. We assume that the project values, *v*, have a triangular distribution ranging from *v*_min_ = 0.25 to *v*_max_ = 1 with a mode at *v*_min_, such that low-value ideas are common and high-value ideas are rare (i.e., *F*(*v*) = 1−(16/9)(1−*v*)^2^). For the cost function, we choose *c*(*v*,*x*) = *x*^2^/*v*. We choose a convex dependence on *x* to suggest that the marginal cost of improving a proposal increases as the proposal becomes stronger. We assume that the intrinsic scientific value of writing a proposal allows investigators to recoup *k* = 1/3 of the disutility cost of proposal preparation. We first explore the case when investigators are motivated purely by the scientific value of their projects (*v*_0_ = 0) and then introduce extrascientific benefits (*v*_0_ = 0.25). In S1 Text and S3–S6 Figs, we provide parallel results with two alternative parameter sets.

The evaluation process by which review panels rank proposals introduces a layer of randomness to the awarding of grants [23–25]. To capture noisy assessment, we use a bivariate copula [26] to specify the joint distribution of a proposal's actual quantile, and its quantile as assessed by the funding agency's review panel. A bivariate copula is a probability distribution on the unit square that has uniformly distributed marginals, as all quantiles must. We use a Clayton copula [27], which allows for accurate assessment of weak proposals, but noisier assessment of strong proposals (S2 Fig). This choice is motivated by the pervasive notion that review panels can readily distinguish strong proposals from weak ones, but struggle to discriminate among strong proposals [16, 25, 28]. A Clayton copula has a single parameter (*θ*) that controls how tightly its two components are correlated. Rather arbitrarily, we use *θ* = 10 in the baseline parameter set. The Clayton copula has the important property that a proposal's probability of funding increases monotonically as its strength increases, regardless of the payline. Thus, we exclude the possibility that panels systematically favor weaker proposals. By using a copula, we implicitly assume that *η*(*x*) depends on *x* only through its rank. In S1 Text, we show how a copula leads to an equation for *ξ*′(*v*), which can then be plugged in to Eq 2.

**Fig 2:**
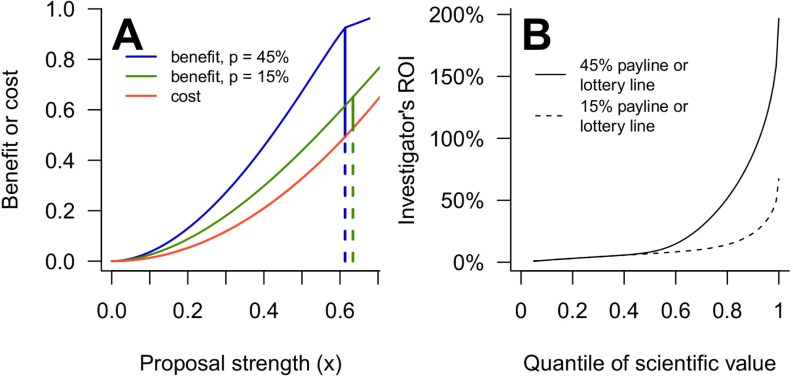
Diminishing paylines reduce investigators' ROIs in a proposal competition. A: Equilibrium benefit (blue or green) and cost (red) curves for an investigator with a project at the 90th percentile of *v* in a proposal competition. The blue and green curves show the benefit at a 45% and 15% payline, respectively. Vertical lines show the investigator's equilibrium bid, with solid portions giving the investigator's payoff and dashed portions showing the cost. The corner in the benefit curves appears at the strongest proposal submitted. B: An investigator's ROI (Eq 3) in a proposal competition with 45% (solid line) or 15% (dashed line) paylines, as a function of the quantile of the scientific value of her project, *F*(*v*). These curves also give the investigator's ROI in a partial lottery with 45% or 15% lottery lines, and any payline. These results use the baseline parameters. ROI, return on investment.

Fig 2 shows numerical results for the baseline parameters at generous (*p* = 45%) and low (*p* = 15%) paylines. In this particular case, investigators' payoffs fall faster than costs as paylines drop, leading to a reduced ROI for everyone at the lower payline (Fig 2B). We will argue below that every investigator's ROI must inevitably fall when the payline becomes small (see S2 and S3 Figs for additional examples).

**Fig 3:**
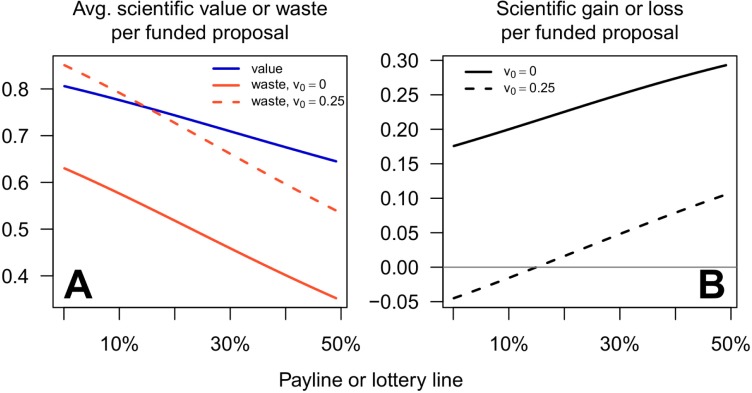
Both decreasing paylines and extrascientific rewards to investigators reduce the scientific efficiency of the funding program. A: Both the average scientific value per funded proposal (blue line, Eq 4) and the average waste per funded proposal (red lines, Eq 5) are higher for lower paylines, for the baseline parameter set and paylines ≤50%. The solid red line shows costs when investigators are motivated only by the scientific value of the funded research (*v*_0_ = 0); the dashed red line shows costs when investigators are additionally motivated by private, extrascientific rewards (*v*_0_ = 0.25). Note that the vertical axis does not extend to 0. B: The scientific gain or loss per funded proposal is lower for lower paylines, both in the absence (*v*_0_ = 0, solid line) and presence (*v*_0_ = 0.25, dashed line) of extrascientific benefits to investigators. Values of other parameters are as specified in the main text. Identical results hold if the horizontal axis is reinterpreted as the proportion of proposals that qualify for a lottery, regardless of the payline. Avg., average.

From the funding agency's perspective, with our baseline parameters, both the average scientific value and average waste per funded proposal increase as the payline falls, for paylines below 50% (Fig 3A). However, as the payline decreases, waste escalates more quickly than scientific value, reducing the scientific gain per funded project (Fig 3B). This same result also appears in our alternative parameter sets (S5 and S6 Figs). We will argue below that the decline in scientific efficiency at low paylines is an inevitable if unfortunate characteristic of proposal competitions.

Clearly, quantitative details of the model's predictions depend on the parameter inputs. To understand the robustness of these predictions, it helps to study the case in which panels discriminate perfectly among proposals. While perfect discrimination is obviously unrealistic in practice, it yields a powerful and general set of results that illuminate how the model behaves when discrimination is imperfect. Numerical results for perfect discrimination under the baseline parameter set appear in S7 and S8 Figs.

At equilibrium under perfect assessment, every project above a threshold value v* = *F*^−1^(1−*p*) will receive funding, and no project idea below this threshold will be funded. Investigators with projects of value *v*>v* all prepare proposals to the identical strength x* = *h*^−1^[(*v*_0_+v*)/((1−*k*)*g*(v*))] and are funded with certainty. Investigators with projects of value *v*<v* opt out (S7 Fig). All of the subsequent results follow (details appear in S1 Text). First, as paylines drop, all investigators realize either a diminishing or zero ROI, because investigators who remain in the competition must pay a higher cost for a reduced payoff. Second, the average scientific value per funded proposal must increase as paylines drop, because only the highest-value projects are funded under low paylines. Third, in the limiting case in which only one of many proposals can be funded (technically, the limit as *p* approaches 0 from above), the scientific value and scientific waste associated with the last funded project converge, and science is no better off than if no grant had been given at all (S8 Fig).

With perfect assessment, there is no general relationship between the scientific efficiency of a proposal competition and the payline that holds across the full range of paylines (but see Hoppe and colleagues [22] for a sharp result when the cost function is independent of *v*). Of course, we wouldn't expect scientific efficiency to decline monotonically with a falling payline, because there are likely to be some low-value projects that can be weeded out at low cost. However, our last result above guarantees that the scientific gain per funded proposal must eventually vanish as the payline declines to a single award.

Returning to the reality of imperfect discrimination, as long as review panels do not systematically favor weaker proposals, noisy assessment changes little about these qualitative results. That is, investigators' ROIs will drop as paylines fall, the average scientific value per funded proposal will increase as paylines decrease, and the scientific efficiency of the proposal competition must eventually decline as the payline approaches a single award. But efficiency need not drop to zero. Perhaps counterintuitively, imperfect discrimination is a saving grace at low paylines. Noisy assessment discourages top investigators from pouring excessive effort into grant writing as paylines fall, because the marginal benefit of writing an even better grant becomes small when review panels struggle to discriminate among top proposals. Indeed, noisy assessment, unlike perfect discrimination, allows a proposal competition to retain a positive impact on science, even with a single funded grant (compare Fig 3 and S8 Fig). This result hints at the salutary nature of randomness at low paylines, which we will see more vividly when we consider lotteries below.

Thus far, we have considered the case in which investigators are motivated only by the scientific value of the projects proposed (*v*_0_ = 0). Now, suppose that investigators are additionally motivated by the extrascientific benefits of receiving a grant, such as professional advancement or prestige (*v*_0_>0). Eq 2 shows that adding extrascientific motivation will increase the effort that investigators devote to preparing grant proposals. However, in our model, at least, this extra effort has no bearing on which grants are funded and thus does not affect the scientific value of the grants that are awarded. Increasing scientific costs without increasing scientific value will clearly be detrimental to the funding program's scientific efficiency. Extrascientific benefits to investigators can even cause the entire funding program to operate at a loss to science when paylines are low (Fig 3).

## Lotteries

Our model can also be used to analyze the efficiency of a partial lottery for advancing science. Suppose that a fraction *q*≥*p* of proposals qualify for the lottery, and each qualifying proposal is equally likely to be chosen for funding. Call *q* the "lottery line." Now, the investigator's payoff is (*p*/*q*)(*v*_0_+*v*)*η*_*l*_(*x*)−(1−*k*)*c*(*v*,*x*), where *η*_*l*_(*x*) is the equilibrium probability that the proposal qualifies for the lottery. In S1 Text, we show that the investigator's bid is given by

$$b(v)=h^{-1}\left[\frac{p}{q}\frac{1}{1-k}\int_{0}^{v}\frac{v_{0}+t}{g(t)}\xi_{l}^{\prime }(t)dt\right]$$ (6)

where *ξ*_*l*_(*v*) = *η*_*l*_(*b*(*v*)).

**Fig 4:**
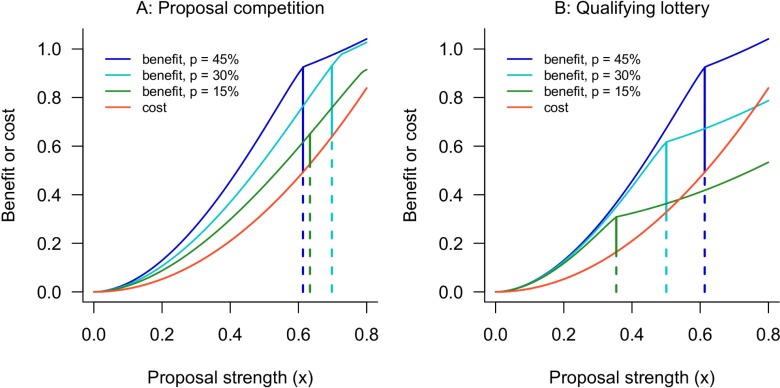
An investigator's ROI falls as the payline drops in a proposal competition but is independent of the payline in a partial lottery. A: Benefit (blue or green) and cost (red) curves for an investigator with a project at the 90th percentile of *v* in a proposal competition. The dark blue, light blue, and green curves show benefits with a 45%, 30%, and 15% payline, respectively. Vertical lines show the investigator's bid, with the length of the solid portion giving the payoff and the length of the dashed portion giving the cost. The investigator's ROI declines as the payline decreases (note also that her effort does not vary monotonically with the payline). B: The same investigator's benefit and cost curves in a partial lottery with a 45% lottery line. The investigator's ROI is the same for all paylines. These results use the baseline parameter set given in the main text. ROI, return on investment.

Our major result for lotteries is that measures of scientific efficiency—expressions 3, 4, and 5—depend on the lottery line *q* but are independent of the payline *p* (proofs appear in S1 Text). This result follows from the fact that, in a lottery, each investigator's benefit and cost are proportional to *p*. Thus, an investigator's ROI and the scientific efficiency of the funding program are determined by the lottery line but are not affected by the payline. To illustrate, Fig 4 compares an investigator's costs and benefits in a proposal competition with 45%, 30%, and 15% paylines versus a partial lottery with a *q* = 45% lottery line and the same three paylines. The key feature of Fig 4 is that the investigator's benefit curve in a partial lottery scales in such a way that her ROI is the same for any payline ≤*q*. Consequently, a partial lottery with a lottery line of *q* and any payline ≤*q* achieves the same scientific efficiency as a proposal competition with a payline of *q*.

Thus, our numerical results showing the investigator's ROI (Fig 2B) or the scientific efficiency (Fig 3) in a funding competition also show the efficiency of a lottery with the equivalent lottery line. That is, a lottery in which 45% of applicants qualify for the lottery has the same scientific efficiency as a proposal competition with a 45% payline, regardless of how many proposals in the lottery are randomly selected for funding. Thus, a lottery can restore the losses in efficiency that a proposal competition suffers as paylines become small.

In S1 Text, we also analyze a more general type of lottery in which proposals are placed into one of a small number of tiers, with proposals in more selective tiers awarded a greater chance of funding [13, 17, 29]. In a multitiered lottery, the efficiency is entirely determined by the number of tiers and the relative probabilities of funding in each, and is independent of the payline. Numerical results (S9 Fig) illustrate that the scientific value and waste of a multitiered lottery fall in between those of a proposal competition and a single-tiered lottery. Thus, a multitiered lottery offers an intermediate design that would partially reduce the waste associated with preparing proposals, while still allowing review panels to reward the best proposals with a higher probability of funding.

## Discussion

Our major result is that proposal competitions are inevitably and inescapably inefficient mechanisms for funding science when the number of awards is smaller than the number of meritorious proposals. The contest model presented here suggests that a partially randomized scheme for allocating funds—that is, a lottery—can restore the efficiency lost as paylines fall, albeit at the expense of reducing the average scientific value of the projects that are funded.

Why does a lottery disengage efficiency from the payline, while a proposal competition does not? For investigators, proposal competitions are, to a first approximation, all-or-nothing affairs, because an investigator only obtains a substantial payoff if her grant is funded. At high paylines (or, more precisely, when the number of awards matches the number of high-value projects), investigators with high-value projects can write proposals that win funding at modest cost to themselves. As the number of awards dwindles, however, competition stiffens. Depending on the details of the assessment process, an investigator with a high-value project must either work harder for the same chance of funding, or work just as hard for a smaller chance of funding. Either way, the return on her investment declines sharply. Thus, a contest is most efficient at the payline that weeds out low-value projects but does not attempt to discriminate among the high-value projects (e.g., S6 Fig). At lower paylines, however, the effort needed to signal which projects are most valuable begins to approach the value of those projects, making the funding program less worthwhile.

In a lottery, investigators do not compete for awards per se, but instead compete for admission to the lottery. The value to the investigator of being admitted to the lottery scales directly with the number of awards. It turns out that both the investigator's expected benefit and her costs of participation scale directly with the payline and thus the payline has no effect on efficiency. (In S1 Text, we follow Hoppe and colleagues [22] to show that this scaling can be explained by the economic principle of revenue equivalence.) If there are fewer awards than high-value projects, a lottery that weeds out the low-value projects but does not attempt to discriminate among high-value projects will facilitate scientific progress more efficiently than a contest.

Unfortunately, empirical comparisons between the efficiencies of funding competitions versus partial lotteries do not yet exist, to the best of our knowledge. However, two recent anecdotes support our prediction that the waste in proposal competitions is driven by the strategic dynamics of the contest itself. First, in 2012, the US National Science Foundation's Divisions of Environmental Biology and Integrative Organismal Systems switched from a twice-per-year, one-stage proposal competition to a once-per-year, two-stage competition, in part to reduce applicants' workload. However, the switch failed to reduce the applicants' aggregate workload meaningfully [30], and the two-stage mechanism was subsequently abandoned. Second, in 2014, the National Health and Medical Research Council of Australia streamlined the process of applying for their Project Grants, cutting the length of an application in half [31]. However, researchers spent more time, not less, preparing proposals after the process had been streamlined, both individually and in aggregate [31]. Both of these experiences are consistent with our prediction that, in a proposal competition, the effort that applicants expend is dictated by the value of funding to the applicants and the number of awards available, but does not depend on the particular format of the proposals.

A lottery is a radical alternative, and may be politically untenable [32]. If a lottery is not viable, an alternative approach to restoring efficiency is to design a contest in which the effort given to competing for awards has more direct scientific value. For example, a contest that rewards good science in its completed form—as opposed to rewarding well-crafted proposals that describe future science—motivates the actual practice of good science, and will be less wasteful at low paylines [9, 14]. Program officers could also be given the discretion to allocate some funds by proactively scouting for promising researchers or projects. Of course, a contest based on completed science or scouting has its own drawbacks, including rich-getting-richer feedback loops, a risk of new barriers to entry for investigators from historically underrepresented demographic groups, and the Goodhart's law phenomenon, whereby a metric that becomes a target ceases to be a good metric [33]. Nevertheless, it is tantalizing to envision a world in which the resources that universities currently devote to helping researchers write proposals are instead devoted to helping researchers do science.

This analysis also shows that extrascientific professional incentives to pursue grant funding can damage the scientific efficiency of a proposal competition. As many of these extrascientific incentives arise from administrators using grant success as a primary yardstick of professional achievement, perhaps one major benefit of adding explicit randomness to the funding mechanism would be to compel administrators to de-emphasize grant success in professional evaluations. Alternatively, to the degree that administrators value and reward grant success because of the associated overhead funds that flow to the university, funding agencies could reduce waste by distributing overhead separately from funding awards. Instead, perhaps overhead could be allocated based partially on the recent past productivity of investigators at qualifying institutions, among other possible criteria. Disengaging overhead from individual grants would encourage administrators to value grants for the science those grants enable (as opposed to the overhead they bring), while allocating overhead based on institutions’ aggregate scientific productivity would motivate universities to help their investigators produce good science.

Funding agencies often have pragmatic reasons to emphasize the meritocratic nature of their award processes. However, our model also suggests that downplaying elements of a funding competition's structure that introduce randomness to funding decisions can increase scientific waste. When applicants fail to recognize the degree to which the contest is already a lottery, they will overinvest effort in preparing proposals, to the detriment of science.

This model does not account for all of the costs or scientific benefits of a proposal competition, including the costs of administering the competition, the time lost to reviewing grant proposals, or the benefit of building scientific community through convening a review panel. Nonetheless, we suggest that the direct value of the science supported by funding awards and the disutility costs of preparing grant proposals are the predominant scientific benefits and costs of the usual proposal competition [13, 30], and provide a useful starting point for a more detailed accounting.

Our model also makes several simplifying assumptions, each of which may provide scope for interesting future work. First, researchers pay a time cost to prepare a proposal but receive money if the proposal is funded. In our model, we have converted both time and money into scientific productivity, in order to place both on a common footing. To be more explicit, though, scientific productivity requires both time and money (among other resources), and researchers may have vastly different needs for both. In S1 Text, we show that our model can be formally extended to encompass researchers' different needs for time and money if the marginal rate of technical substitution (that is, the rate at which time and money can be exchanged without altering scientific productivity) is exactly correlated with the project's scientific value. Our main results still hold in this case, as long as researchers with the best ideas do not value time so greatly that they write the weakest proposals. A more general exploration of researchers' heterogeneous needs for time and money—and of how researchers may adjust their portfolio of scientific activities when time or money is scarce—provide ample opportunity for future work.

Second, our model assumes that the distribution of the scientific value (*v*) across possible projects is exogeneous to the structure of the funding competition. This may not be the case if, for instance, a partial lottery encourages participation by investigators with unconventional views, reduces the psychological stigma of previous rejection [16], or discourages investigators either who have succeeded under the traditional proposal competition format or who perceive a lottery as riskier. In reality, such feedback loops may endogenize the distribution of *v*. Third, our model does not consider the savings that may accrue to investigators if they can submit a revised version of a rejected proposal to a different or subsequent competition. To a first approximation, submissions to multiple funders have the effect of increasing *p*, which can then be interpreted more generally as the proportion of ideas that get funded across all available funding programs. Iterations of revision and resubmission to the same funding program are likely to have more complex effects on efficiency and waste. Finally, our model is silent regarding whether many small or few large grants will promote scientific progress most efficiently, and is likewise silent about the factors that will influence this comparison.

To be sure, much more can be done to embellish this model. However, the qualitative results—that proposal competitions become increasingly inefficient as paylines drop and that professional pressure on investigators to pursue funding exacerbates these inefficiencies—are inherent to the structure of contests. Partial lotteries and contests that reward past success present radical alternatives for allocating funds and are sure to be controversial. Nevertheless, whatever their other merits and drawbacks, these alternatives could restore efficiency in distributing funds that has been lost as those funds have become increasingly scarce.

## Supporting information

S1 Fig The success rate of grant proposals for NIH R01 and equivalents has declined substantially over the past 50 years. Data from FY 1962–2008 include R01, R23, R29, and R37 proposals, as reported by NIH's Office of Extramural Research [34]. Data from 1962–1969 are NIH estimates. Data for FY 2009–2016 include R01 and R37 proposals, as reported by [2] (R01 and R37 provide the vast majority of proposals for earlier years). Data include new applications, supplements, and renewals, and the success rate is calculated as the number of proposals funded divided by the number of proposals reviewed. FY, fiscal year; NIH, National Institutes of Health.
(TIF)

S2 Fig Random samples from copula distributions used to model error in assessment of grant proposals. A: Clayton copula with *θ* = 10. B: Clayton copula with *θ* = 5. Blue dashed lines give the median of the assessed quantile as a function of the actual quantile.
(TIF)

S3 Fig Parallel results to Fig 2, except with the first alternative parameter set. See S1 Text for parameter values in this alternative parameter set.
(EPS)

S4 Fig Parallel results to Fig 2, except with the second alternative parameter set. See S1 Text for parameter values in this alternative parameter set.
(EPS)

S5 Fig Parallel results to Fig 3, except with the first alternative parameter set. Note that the vertical axis in panel A does not extend to 0.
(EPS)

S6 Fig Parallel results to Fig 3, except with the second alternative parameter set. In this figure, data are shown for paylines ranging from *p* = 0.001 to *p* = 0.999. Note that the vertical axis in panel A does not extend to 0.
(EPS)

S7 Fig Parallel results to Fig 2, except with perfect assessment of proposal strength. All other parameter values are the same as in Fig 2.
(EPS)

S8 Fig Parallel results to Fig 3, except with perfect assessment of proposal strength. All other parameter values are the same as in Fig 3. Note that the vertical axis in panel A does not extend to 0.
(EPS)

S9 Fig Parallel results to Fig 3 for a three-tier lottery with equally sized tiers and a 3:2:1 ratio of funding probabilities across the tiers. The horizontal axis gives the proportion of proposals that qualify for any tier of the lottery. Scientific value and scientific waste per funded proposal are independent of the actual payline, as long as the payline is less than 2/3 of the lottery line. (If the payline exceeds 2/3 of the lottery line, then the ratios of funding across tiers will be something other than 3:2:1, and thus the average value and average cost of a funded proposal will change slightly.) All other parameter values are the same as in Fig 3. Note that the vertical axis in panel A does not extend to 0.
(EPS)

S1 Text Mathematical details. (PDF)

## Abbreviation

ROI: return on investment
