# Supplementary figures and images for: Contest models highlight inherent inefficiencies of scientific funding competitions
Source: PLoS Biol. 2019 Jan 2;17(1):e3000065. doi: 10.1371/journal.pbio.3000065 (PMC6314589; doi:10.1371/journal.pbio.3000065)

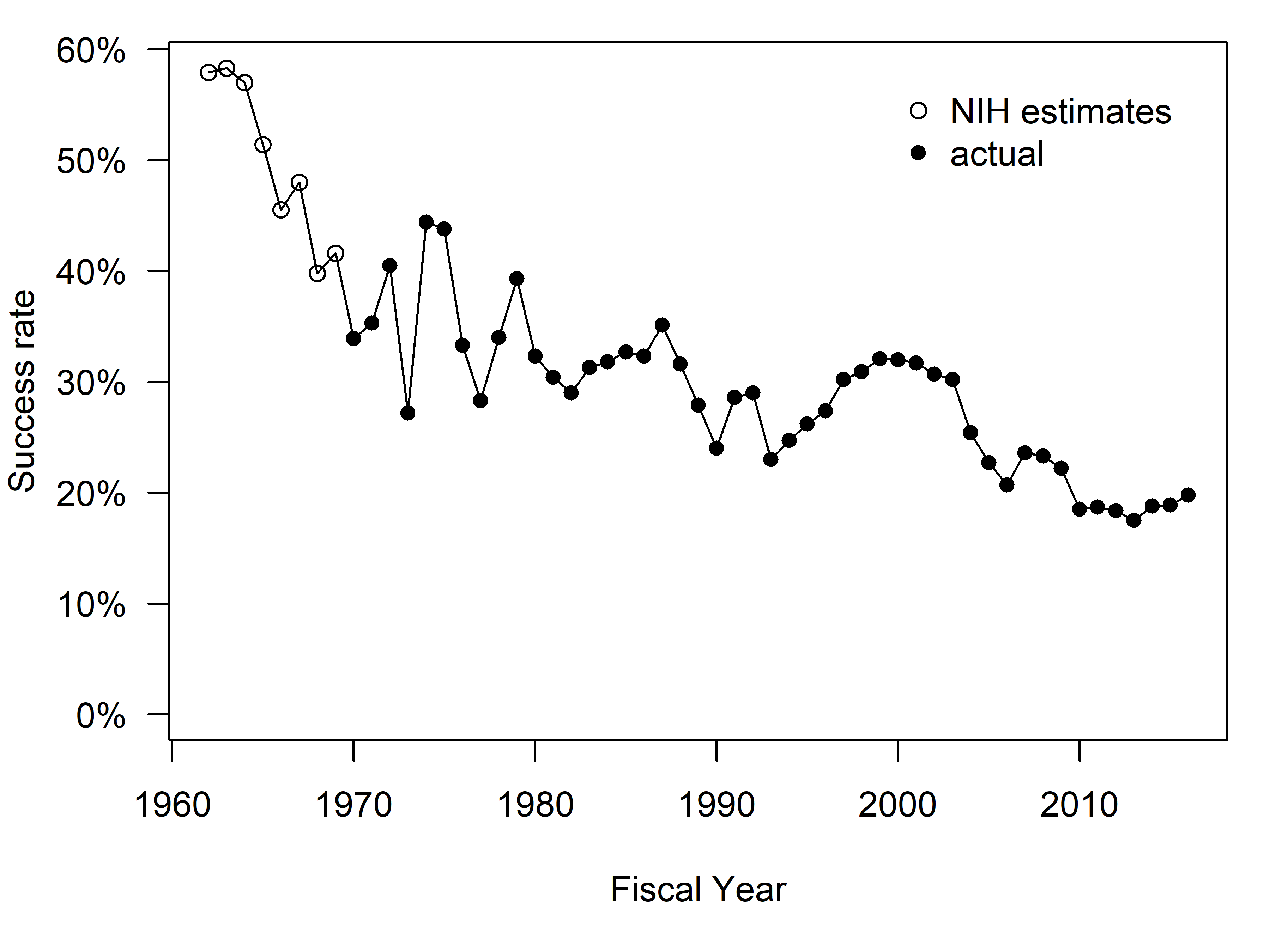

Supplement: S1 Fig — Data from FY 1962–2008 include R01, R23, R29, and R37 proposals, as reported by NIH's Office of Extramural Research [34]. Data from 1962–1969 are NIH estimates. Data for FY 2009–2016 include R01 and R37 proposals, as reported by [2] (R01 and R37 provide the vast majority of proposals for earlier years). Data include new applications, supplements, and renewals, and the success rate is calculated as the number of proposals funded divided by the number of proposals reviewed. FY, fiscal year; NIH, National Institutes of Health. (TIF) [file pbio.3000065.s001.tif]

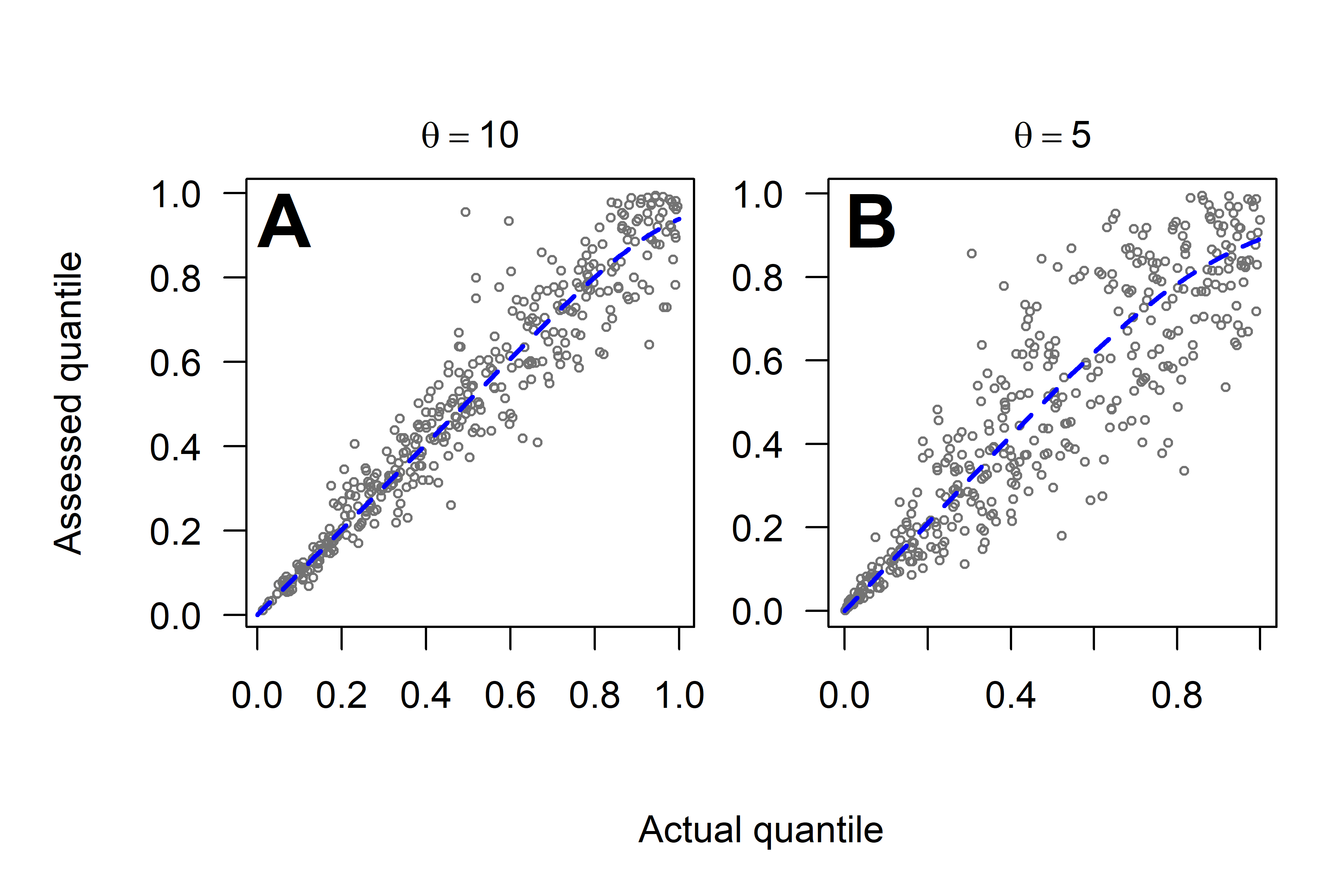

Supplement: S2 Fig — A: Clayton copula with θ = 10. B: Clayton copula with θ = 5. Blue dashed lines give the median of the assessed quantile as a function of the actual quantile. (TIF) [file pbio.3000065.s002.tif]
