# Supplementary material for: Contest models highlight inherent inefficiencies of scientific funding competitions
Source: PLoS Biol. 2019 Jan 2;17(1):e3000065. doi: 10.1371/journal.pbio.3000065 (PMC6314589; doi:10.1371/journal.pbio.3000065)
Supplement: S1 Text — (PDF) [file pbio.3000065.s010.pdf]

## S1 TEXT: MATHEMATICAL PROOFS AND DERIVATIONS

### *Bid function*

The bid function $b(v)$ can be found following steps that are identical to the derivation of the first part of Proposition 8 in Hoppe *et al.* [22]. We repeat that derivation here, almost fully verbatim.

Begin by dividing the investigator's payoff function by by $(1-k)g(v)$ to rescale the investigator's optimization problem (eq. 1) :

$$b(v) = \arg\max_x \left\{ \frac{(v_0 + v)}{(1-k)g(v)} \eta(x) - h(x) \right\}. \tag{S1}$$

Consider two projects of values $v_1$ and $v_2$, $v_1 > v_2$, with equilibrium bids $b(v_1)$ and $b(v_2)$, respectively. The investigator with a project of value $v_1$ should not submit a bid as if her project had value $v_2$, and similarly the investigator whose project has value $v_2$ should not submit a bid as if her project had value $v_1$. This yields:

$$\frac{(v_0+v_1)}{(1-k)g(v_1)}\eta(b(v_1)) - h(b(v_1)) \geq \frac{(v_0+v_1)}{(1-k)g(v_1)}\eta(b(v_2)) - h(b(v_2))$$

$$\frac{(v_0+v_2)}{(1-k)g(v_2)}\eta(b(v_2)) - h(b(v_2)) \geq \frac{(v_0+v_2)}{(1-k)g(v_2)}\eta(b(v_1)) - h(b(v_1)).$$

Rearrange each inequality to isolate $h(b(v_1)) - h(b(v_2))$ and divide through by $v_1 - v_2$ to give

$$\frac{\frac{(v_0+v_2)}{(1-k)g(v_2)}(\eta(b(v_1)) - \eta(b(v_2)))}{v_1 - v_2} \leq \frac{h(b(v)) - h(b(\hat{v}))}{v_1 - v_2} \leq \frac{\frac{(v_0+v_1)}{(1-k)g(v_1)}(\eta(b(v_1)) - \eta(b(v_2)))}{v_1 - v_2}.$$

Take the limit as $v_2 \to v_1$ to give

$$\frac{d}{dv}h(b(v)) = \frac{v_0 + v}{(1-k)g(v)} \frac{d}{dv}\eta(b(v)). \tag{S2}$$

Essentially, after rescaling investigators' benefits and costs so that the cost function ($h(x)$) is the same for all investigators, eq. S2 says that, at equilibrium, an investigator's marginal (re-scaled) cost and marginal (re-scaled) benefit of preparing an infinitesimally stronger proposal are equal [22]. Proceeding with the derivation, multiply both sides of eq. S2 by $dv$ to separate variables and integrate from 0 to $v$ to obtain

$$\int_0^v dh(b(t)) = \int_0^v \frac{v_0 + t}{(1-k)g(t)}\, \xi'(t)\, dt \tag{S3}$$

where $\xi(v) = \eta(b(v))$. Then use $h(b(0)) = h(0) = 0$ to give $\int_0^v dh(b(t)) = h(b(v))$, and thus

$$h(b(v)) = \int_0^v \frac{v_0 + t}{(1-k)g(t)}\, \xi'(t)\, dt. \tag{S4}$$

Take $h^{-1}$ on both sides to complete the derivation.

To make our derivation somewhat more general than in the main text, suppose that both $v_0$ and $k$ are themselves functions of $v$, such that the investigator's optimization problem now becomes

$$b(v) = \arg\max_x \left\{ \frac{(v_0(v) + v)}{(1-k(v))g(v)} \eta(x) - h(x) \right\}. \tag{S5}$$

We require the key condition that the quantity $\frac{(v_0(v)+v)}{(1-k(v))g(v)}$ is a strictly increasing function of $v$, so that investigators with higher value projects will submit stronger proposals. This condition rules out the possibility that, say, variation in $v_0$ or $k$ is large enough that either replaces $v$ as the primary correlate of proposal strength. When this key condition holds, the derivation above proceeds as before, leading to a bid function of

$$b(v) = h^{-1}\left[\int_0^v \frac{v_0(t) + t}{(1-k(t))g(t)}\, \xi'(t)\, dt\right]. \tag{S6}$$

In this case, variation in $v_0$ or $k$ among investigators may change the value of investigators' bids, but it does not change the rank of investigators' bids (that is, higher-value projects are still associated with stronger proposals). Because we use a copula to capture noisy assessment of proposals, an investigator's probability of funding depends only on the rank of the investigator's bid, and thus the portfolio of funded projects does not change.

Finally, we can extend the model to accommodate researchers' differing needs for time and money, as long as the marginal rate of technical substitution for time vs. money is also a function of $v$. In this case, we re-interpret the cost function $c(v, x) = g(v)h(x)$ as the time cost of writing a proposal, and we assume that the monetary cost of writing a proposal is negligible. Let $\phi$ be a conversion factor that converts time into scientific productivity, such that the disutility cost of writing a proposal in terms of lost productivity is $\phi c(v, x)$. Moreover, suppose $\phi$ is also a function of $v$. Then, we can write the investigator's optimization problem as

$$b(v) = \arg\max_x \left\{(v_0(v) + v)\eta(x) - (1-k(v))\phi(v)g(v)h(x)\right\}. \tag{S7}$$

As before, we require the key condition that $\frac{(v_0(v)+v)}{(1-k(v))\phi(v)g(v)}$ is a strictly increasing function of $v$, to ensure that proposal strength is positively correlated with scientific value at equilibrium. Under this condition, the same steps give a bid function of

$$b(v) = h^{-1}\left[\int_0^v \frac{v_0(t)+t}{(1-k(t))\phi(t)g(t)}\,\xi'(t)\,dt\right]. \qquad \text{(S8)}$$

To demonstrate that $b(v)$ maximizes the investigator's payoff (as opposed to minimizing it), we follow Moldovanu & Sela's [20] "pseudo-concavity" argument. This argument requires that $b'(v) > 0$, which we establish first. To do so, note that $h^{-1}$ in eq. 2 is an increasing function (because $h$ is an increasing function), and that $(v_0 + t)/g(t) > 0$ in the integrand of eq. 2. Thus, to show that $b'(v) > 0$, it suffices to show that $\xi'(v) > 0$, that is, that the probability of being funded increases as the value of the scientific project increases. We expect this condition to hold under any reasonable model of how proposals are assessed. Basic but tedious calculus establishes that it does hold for the Clayton copula that we describe below.

Having established that $b'(v) > 0$, the pseudo-concavity argument of Moldovanu & Sela [20] proceeds as follows. Let $\varpi(v,x) = (v_0+v)\eta(x) - (1-k)g(v)h(x)$ be the payoff associated with a project of value $v$ and a proposal of quality $x$. Let $\varpi_x = \partial\varpi(v,x)/\partial x$. We claim that $\varpi_x > 0$ for $x < b(v)$, and $\varpi_x < 0$ for $x > b(v)$. These claims, together with the continuity of $b(v)$, establish that $x = b(v)$ maximizes $\varpi(v,x)$.

We first show that $\varpi_x > 0$ for $x < b(v)$. Choose a value of $x < b(v)$, and let $v^\star$ be the value of a project that will generate a bid of $x$, that is, $b(v^\star) = x$. Because $b'(v) > 0$, it follows that $v^\star < v$. Simple differentiation gives

$$\varpi_x(v,x) = (v_0+v)\eta'(x) - (1-k)g(v)h'(x). \qquad \text{(S9)}$$

Now differentiate $\varpi_x$ with respect to $v$ to give the mixed derivative

$$\varpi_{xv}(v,x) = \eta'(x) - (1-k)g'(v)h'(x). \qquad \text{(S10)}$$

Under the assumptions of our model, $\eta'(x) > 0$, $g'(v) < 0$, and $h'(x) > 0$; thus, $\varpi_{xv} > 0$. Therefore, $\varpi_x$ is an increasing function of $v$, and thus $\varpi_x(v^\star,x) < \varpi_x(v,x)$. By virtue of the fact that $b(v^\star) = x$, we have $\varpi_x(v^\star,x) = 0$. Therefore, $\varpi_x(v,x) > 0$.

The proof that $\varpi_x < 0$ for $x > b(v)$ follows similarly.

### *Copulas for noisy assessment*

A bivariate copula is simply a bivariate probability distribution on the unit square with uniform marginal distributions [26]. Let $U = F(v)$ be the actual quantile of a proposal, and let $W$ be the assessed quantile. The joint distribution of $U$ and $W$ is given by the copula $\mathcal{C}(u,w) = \Pr\{U \le u, W \le w\}$. Given a value of $U$, the conditional distribution of $W$ given $U$ is $\mathcal{C}_{W|U}(u,w) = \Pr\{W \le w | U = u\} = \partial\mathcal{C}(u,w)/\partial u$. (Here we use the fact that $U$ is uniformly distributed on the unit interval.) To find $\xi(v)$, evaluate $\mathcal{C}_{W|U}$ at $u = F(v)$ and $w = 1-p$ to find $1-\xi(v)$, the probability that an idea of value $v$ is not funded. Take the complement to find $\xi(v)$. Differentiate with respect to $v$ to find $\xi'(v)$, which can then be plugged in to eq. 2.

The distribution function for a Clayton copula [27] is [26, §4.2]

$$\mathcal{C}(u,w) = \left(u^{-\theta} + w^{-\theta} - 1\right)^{-1/\theta}. \qquad \text{(S11)}$$

The parameter $\theta \ge 0$ controls the strength of the association between $U$ and $W$, with larger values of $\theta$ giving stronger associations (i.e., more accurate assessment of grant proposals).

### *Alternative parameter sets*

To complement the example in the main text, we show numerical results for two alternative parameter sets. In the first alternative set, scientific value is uniformly distributed across projects, the disutility cost increases linearly with proposal quality, and assessment is less precise than we assume in the baseline parameter set. In this set, $v$ is uniformly distributed between 1/3 and 1. We use $c(v,x) = xe^{-v}$ for the cost function, and we use $\theta = 5$ in the Clayton copula (Fig. S2B).

The second alternative parameter set captures a scenario where the pool of possible project values is bimodal, with many minimal-value projects and equally many maximal-value projects. In this alternative set, $v$ ranges from 1/2 to 1. To construct the distribution of $v$, let $Y$ be a beta random variable with both shape parameters equal to 1/2. Thus, $Y$ has a symmetric, U-shaped distribution on the unit interval. Then $v$ is given by $(1+Y)/2$. In this parameter set, we choose $c(v,x) = (1.5-v)x^2$, and we set $\theta = 7.5$ in the Clayton copula.

Both alternative parameter sets use $k = 1/3$.

### *Perfect discrimination*

In the perfect discrimination case, we require that there is a maximum possible value of $v$, which we write $v_{\max}$. The previous derivation of the bid function does not work for perfect discrimination, because $b(v)$ becomes a step function and thus is not differentiable. Instead, let $v^\star$ denote the threshold value, that is, $v^\star = F^{-1}(1-p)$. Under perfect discrimination, the threshold investigator

will break even regardless of her bid. Thus,

$$\eta(x) = \begin{cases} \dfrac{g_k(v^\star)h(x)}{v_0 + v^\star} & x \le x^\star \\ 1 & x > x^\star \end{cases} \tag{S12}$$

where

$$x^\star = h^{-1}\left[\frac{v_0 + v^\star}{g_k(v^\star)}\right]. \tag{S13}$$

Consequently, it is straightforward to show that the bid function is

$$b(v) = \begin{cases} x^\star & v > v^\star \\ 0 & v < v^\star. \end{cases} \tag{S14}$$

The following results are all immediate. First, as the payline drops, $v^\star$ increases, and hence $x^\star$ increases. (Recall that $g$ is a strictly decreasing function, and $h^{-1}$ is a strictly increasing function.) Thus, investigators with $v > v^\star$ experience a reduced payoff and increased costs, leading to a reduced ROI. Second, the average scientific benefit per funded proposal, which can be written as

$$\int_{v^\star}^{v_{\max}} v\,dF(v) \Big/ \int_{v^\star}^{v_{\max}} dF(v), \tag{S15}$$

increases as $v^\star$ increases. Third, as $p$ approaches 0 from above, $v^\star$ approaches $v_{\max}$ from below. Thus, in the limit, the bid function approaches

$$\lim_{p\to 0^+} b(v) = \begin{cases} h^{-1}\left[\dfrac{v_0 + v_{\max}}{g_k(v_{\max})}\right] & v = v_{\max} \\ 0 & v < v_{\max}. \end{cases} \tag{S16}$$

Thus, the payoff to all investigators approaches 0 as $p$ approaches 0 from above.

### *Lotteries*

We consider the more general case of a multi-tier lottery. Proposals deemed worthy of funding are placed into one of $z$ tiers, with tier 1 representing the highest-ranked proposals, etc. Write the proportion of proposals in tier $i$ as $q_i$, and let $\pi_i$ represent the probability that a proposal placed in tier $i$ is funded, where $1 \ge \pi_1 > \pi_2 > \ldots > \pi_z > 0$. We assume that the funding agency determines $q_1, q_2, \ldots, q_z$ and $\pi_1, \pi_2, \ldots, \pi_z$ in advance. Because the payline is still $p$, we must have $\sum_{i=1}^{z} q_i\pi_i = p$. The single-tier lottery proposed by Fang & Casadevall [16] and others is a special case with $z = 1$, with a probability of funding $\pi_1 = p/q_1$ in that tier.

In a tiered lottery, the investigator's maximization problem becomes

$$b(v) = \arg\max_x \left\{ (v_0 + v)\sum_{i=1}^{z} \pi_i\eta_i(x) - (1-k)c(v,x) \right\} \tag{S17}$$

where $\eta_i(x)$ is the probability that a proposal of quality $x$ is placed in tier $i$. A similar derivation to the steps in eq. S2–S4 yields the bid function

$$b(v) = h^{-1}\left[\sum_{i=1}^{z} \frac{\pi_i}{1-k}\int_0^v \frac{v_0 + t}{g(t)}\,\xi_i'(t)\,dt\right]. \tag{S18}$$

where $\xi_i(v) = \eta_i(b(v))$.

We now show that the efficiency of a lottery depends entirely on the structure of the lottery, and is independent of the payline. For multi-tier lotteries, we require that the ratios of the $\pi_i$'s — the probabilities of funding in each tier — are fixed. To establish these ratios, write $\kappa_i = \pi_i/\pi_1$. The condition $\sum_{i=1}^{z} q_i\pi_i = p$ implies that the probability that a proposal in tier $i$ is funded is

$$\pi_i = \frac{p\kappa_i}{\sum_i \kappa_i q_i}, \tag{S19}$$

as long as $p \le \sum_i \kappa_i q_i$. (If $p > \sum_i \kappa_i q_i$, then we would have $\pi_1 > 1$.)

All of our results follow from showing that an investigator's benefit and cost are proportional to $p$, and thus the payline $p$ cancels out of the efficiency calculations in eqq. 3–5. The investigator's benefit from entering the competition is $(v_0 + v)\sum_{i=1}^{z} \pi_i\eta_i(x)$. A simple substitution shows that this benefit is proportional to $p$:

$$\begin{aligned}(v_0 + v)\sum_{i=1}^{z}\pi_i\eta_i(x) &= (v_0 + v)\sum_{i=1}^{z}\frac{p\kappa_i\eta_i(x)}{\sum_j \kappa_j q_j} \\ &= p(v_0 + v)\frac{\sum_i \eta_i(x)\kappa_i}{\sum_j \kappa_j q_j}.\end{aligned}$$

To show that the investigator's cost is proportional to $p$, we have

$$\begin{aligned}c(v, b(v)) &= g(v)h(b(v)) \\ &= g(v)\sum_{i=1}^{z}\frac{\pi_i}{1-k}\int_0^v \frac{v_0 + t}{g(t)}\,\xi_i'(t)\,dt \\ &= p\,g(v)\frac{\sum_i \kappa_i \int_0^v \frac{v_0+t}{g(t)}\,\xi_i'(t)\,dt}{(1-k)\sum_j \kappa_j q_j}.\end{aligned}$$

It thus follows that the investigator's ROI (eq. 3), the average value per funded grant (eq. 4), and the average waste per funded grant (eq. 5) are all independent of $p$.

Hoppe *et al.* [22] provide an argument based on the economic principle of revenue equivalence that explains why costs are independent of the payline in a lottery. This argument applies both to proposal competitions and to lotteries of any structure, and it applies regardless of whether panels discriminate perfectly among proposals, or not. The argument is most easily explained in a single-tier lottery with perfect discrimination, so we consider that setting. First, for revenue equivalence to apply, we need to re-scale the model so that only benefits vary among investigators. That is, re-scale the investigator's equilibrium benefit function to

$$\frac{p}{q}\frac{(v_0 + v)}{(1-k)g(v)}\xi_l(v) \tag{S20}$$

and write her cost function as $h(x)$. Having re-scaled the investigator's benefits and costs, the principle of revenue equivalence suggests that the (re-scaled) cost paid by an investigator will be exactly equal to the negative externality that her entrance into the competition creates, that is, the amount by which her entrance decreases the aggregate benefit of the competing investigators [22]. With perfect discrimination, the threshold investigator (the last one to qualify for the lottery) has project value $v^\star = F^{-1}(1-q)$; every investigator with $v > v^\star$ qualifies for the lottery, while every investigator with $v < v^\star$ opts out. Consider an investigator with an idea of value $v$. If $v < v^\star$, then this investigator's entrance into the competition has no effect on other investigators' benefit, and thus the cost she pays is 0 (i.e., she opts out). If the investigator has an idea of value $v > v^\star$, then she knocks one threshold investigator out of the lottery. No other investigator's benefit changes. Thus, the new investigator's presence decreases the aggregate benefit of the other investigators by

$$\frac{p}{q}\frac{(v_0 + v^\star)}{(1-k)g(v^\star)}. \tag{S21}$$

This negative externality is exactly the re-scaled cost that she pays, i.e.,

$$h(b(v)) = \frac{p}{q}\frac{(v_0 + v^\star)}{(1-k)g(v^\star)}. \tag{S22}$$

Multiplying by $g(v)$ undoes the re-scaling to give the actual cost paid:

$$c(v, b(v)) = g(v)h(b(v)) = g(v)\frac{p}{q}\frac{(v_0 + v^\star)}{(1-k)g(v^\star)}. \tag{S23}$$

Two observations explain why the cost paid by the investigator is directly proportional to $p$. First, the identity of the threshold investigator — the one who is knocked out of the lottery when an investigator with a higher-value project enters — is determined by $q$, not $p$. (In a proposal competition, the threshold investigator is determined by $p$.) Second, the threshold investigator's benefit — and hence the negative externality imposed by the newly arriving investigator — is directly proportional to $p$. Thus, the (re-scaled) cost paid by any investigator will also be directly proportional to $p$. Multiplying the cost by $g(v)$ to undo the rescaling does not change the direct proportionality to $p$.

With noisy assessment, and/or in a multi-tiered lottery, the negative externality that an investigator imposes on the field, and hence the (re-scaled) cost that she pays at equilibrium, integrates the amount by which her entry decreases the benefit of every investigator with a project value less than hers. (This is one way to understand the bid functions in eq. 2, 6, and S18.) In a lottery, everyone's benefit is proportional to $p$, and hence each investigator's negative externality, and the cost that she pays, is proportional to $p$ as well.
